# Supplementary material for: Bariatric Surgery Induced Changes in Blood Cholesterol Are Modulated by Vitamin D Status
Source: Nutrients. 2022 May 10;14(10):2000. doi: 10.3390/nu14102000 (PMC9145606; doi:10.3390/nu14102000)
Supplement: Supplementary file 1 [file nutrients-14-02000-s001.zip › nutrients-1696417-supplementary.pdf]

Supplement Table S1. Intervention results for biochemistry parameters adjusted to baseline values of BMI and body weight.

Covariant	BMI		Body weight	
ANCOVA	p	$\eta 2p \eta p2$	p	$\eta 2p \eta p2$
hsCRP mg/L]	*0.01*	*0.18*	*0.01*	*0.23*
LDL [mg/dL]	0.81	0.01	0.80	0.01
HDLC[mg/dL]	0.10	0.07	0.04	0.10
CHOL[mg/dL]	0.10	0.07	0.07	0.08
TG [mg/dL]	0.10	0.07	0.06	0.09
GLU [mg/dL]	0.00	0.26	0.00	0.27
ALB [g/L]	0.01	0.17	0.00	0.25
PRO [g/L]	0.09	0.08	0.02	0.13

Note: hsCRP, C-reactive protein highly sensitive; LDLC, low-density lipoprotein cholesterol; HDLC, high-density lipoprotein cholesterol; CHOL, cholesterol; TG, triglycerides; GLU, glucose; ALB, albumin; PRO, protein; ANCOVA, analysis of covariance.

Supplement Table S2. Correlation between baseline vitamin D and metabolic parameters.

Baseline	Weight (B)	Weight (C)	BMI (B)	BMI (C)
25(OH)D3 [ng/mL]	−0.07	−0.12	−0.05	0.11
25(OH)D2 [ng/mL]	−0.10	−0.17	−0.12	−0.23
24,25(OH)2D3 [ng/mL]	−0.04	−0.10	−0.08	0.18
epi-25(OH)D3 [ng/mL]	−0.18	−0.13	−0.10	0.06
